# FAM20C Modulates Neuronal Differentiation in Hypoxic–Ischemic Brain Damage via KAP1 Phosphorylation and LINE1 RNA m6A‐Dependent H3K9me3 Regulation

**DOI:** 10.1111/cpr.70073

**Published:** 2025-06-13

**Authors:** Chen‐xi Feng, Mei Wang, Gen Li, Si‐jia Chu, Di Wu, Xiao‐han Hu, Li‐xiao Xu, Mei Li, Xing Feng

**Affiliations:** ^1^ Institute of Pediatric Research Children's Hospital of Soochow University Suzhou China; ^2^ Department of Pharmacy Children's Hospital of Soochow University Suzhou China; ^3^ Department of Neonatology Children's Hospital of Soochow University Suzhou China

**Keywords:** extracellular serine/threonine protein kinase FAM20C, hypoxic–ischemic brain damage (HIBD), neurodevelopmental impairment

## Abstract

Neurodevelopmental impairment due to hypoxic–ischemic brain damage (HIBD) lacks effective biomarkers and therapeutic targets. Based on some cues from published papers, extracellular serine/threonine protein kinase FAM20C was speculated to play a crucial role in the neurodevelopmental impairment of HIBD. In this study, FAM20C was found suppressed in the ischemic hippocampal tissue of HIBD. The inhibition of FAM20C caused by HIBD affected cell differentiation and subsequently caused cognitive impairment. KAP1 was identified as a kinase substrate of FAM20C in the central nervous system. The regulation of the YTHDC1‐NCL‐KAP1‐LINE1 RNA complex by FAM20C was mediated through KAP1 phosphorylation and LINE1 RNA m6A. These alterations consequently modulated the establishment of the H3K9me3 modification on LINE1 DNA, thereby resulting in neuronal differentiation. Furthermore, E2F4, identified as a transcription factor, regulated FAM20C in HIBD. This research has clarified the novel association between FAM20C and HIBD, laying the foundation for innovative diagnostic and therapeutic strategies to counteract neurodevelopmental disruptions arising from neonatal hypoxic–ischemic encephalopathy (HIE).

## Graphical abstract

FAM20C regulates the composition of the YTHDC1‐NCL‐KAP1‐LINE1 RNAs complex, thereby affecting neuronal differentiation in hypoxic–ischemic brain damage (HIBD).

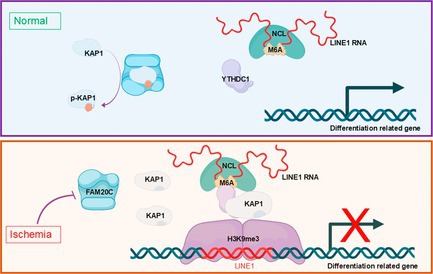

**Abbreviations**
ADAR: Adenosine deaminase RNA specific
AFP: Alpha fetoprotein
APOE: Apolipoprotein E
CK1: Casein kinase 1/cell kinase 1
CK2: Casein kinase 2/cell kinase 2
Co‐IP: Co‐immunoprecipitation
CP: Ceruloplasmin
DAGLA: Diacylglycerol lipase‐alpha
E2F4: E2F transcription factor 4
EDTA: Extensive de novo TE annotator
ELISA: Enzyme linked immunosorbent assay
FAM20C: Extracellular serine/threonine protein kinase FAM20C
FOXK2: Forkhead box K2
GFAP: Glial fibrillary acidic protein
HIBD: Hypoxic–ischemic brain damage
HIE: Hypoxic–ischemic encephalopathy
HIF‐1α: Hypoxia inducible factor 1 subunit alpha
IBA1: Ionised calcium‐binding adapter molecule 1
IP: immunoprecipitation
KAP1: KRAB‐associated protein 1
LINE: Long interspersed nuclear elements
LTR: Long terminal repeat‐retrotransposons
MWM: Morris water maze
NeuN: Neuronal nuclei antigen
NOR: Novel object recognition
NSCs: Neural stem cells
Phos‐WB: Phos‐tag Western blot
PTK2B: Protein‐tyrosine kinase 2‐beta
SAFB: Scaffold attachment factor
SINE: Short interspersed nuclear elements
SQSTM1: Sequestosome 1
YTHDC1: YTH domain‐containing protein 1
ZNF740: Zinc finger protein 740

## 1 Background

Neonatal hypoxic–ischemic brain damage (HIBD), caused by perinatal asphyxia or hypoxia, leads to high mortality and severe sequelae. About 30% of survivors of HIBD experience neurodevelopmental problems, such as cerebral palsy, epilepsy and intellectual disabilities. Currently, there is a lack of specific biomarkers and therapeutic targets for the diagnosis of neurodevelopmental impairment caused by HIBD. By re‐analysing data from a published paper [1], four potential biomarkers for distinguishing the severity of hypoxic–ischemic encephalopathy (HIE) have been found. It has been reported that three of them are specific phosphorylated substrates of extracellular serine/threonine protein kinase FAM20C (FAM20C) [2], which controls substrate secretion via phosphorylation‐dependent mechanisms [3]. These findings led us to hypothesise that FAM20C might regulate the abnormal levels of APOE, CP and AFP observed in HIE blood samples. Taken together, the role of FAM20C in HIBD has garnered attention.

Protein phosphorylation signalling pathways mediated by serine/threonine protein kinases are prevalent across diverse tissues and are implicated in cellular processes such as proliferation, differentiation, transformation and apoptosis. Studies have documented the role of serine/threonine protein kinases in modulating neuronal survival and synaptic function, influencing the progression of a spectrum of neurological diseases [4]. FAM20C, also known as dentin matrix protein 4, is a member of the serine/threonine kinase family and can phosphorylate proteins with the Ser‐x‐Glu/pSer motif. The non‐lethal manifestation of Raine syndrome caused by FAM20C deficiency presents neurodevelopmental impairment, including developmental delays, intellectual disabilities, hearing loss and seizures, which are exacerbated with the aging and maturation of the central nervous system. Computational predictions indicate that target proteins of FAM20C in brain tissue and cerebrospinal fluid are involved in axonal transport and neuronal components [5]. Based on these clues, we hypothesised that FAM20C influences neurodevelopment.

In this study, we concentrated on the implications of FAM20C for neurodevelopmental disorders within HIBD. We established a rat model of HIBD and detected changes in the expression and enzymatic activity of FAM20C in the hippocampus. We explored the influence of FAM20C on neurodevelopmental impairment caused by HIBD, with cellular assays and behavioural evaluations. Using protein‐strip mass spectrometry and molecular biology experiments, we identified a specific phosphorylated substrate of FAM20C in hippocampal tissue, and we probed into the downstream molecules and pathways potentially modulated by FAM20C. Subsequently, through proteomic analyses, transcription factor profiling, and molecular biology methodologies, we identified the transcription factor, E2F transcription factor 4 (E2F4), as a critical regulator potentially mediating FAM20C downregulation in HIBD.

## 2 Results

### 2.1 FAM20C Is Inhibited in the Ischemic Hippocampal Tissue After HIBD

This study reanalyzed previous neonatal asphyxia data using weighted gene co‐expression network analysis (WGCNA) and principal component analysis (PCA). Four biomarkers were found: ceruloplasmin (CP), apolipoprotein E (APOE), protein‐tyrosine kinase 2‐beta (PTK2B), alpha fetoprotein (AFP) (Figure S1) and CP, APOE and AFP. ELISA results demonstrated significant changes of CP, APOE and AFP, which are reported as specific serine/threonine kinase substrates of FAM20C in the serum of severe HIBD rats (Figure S1). These results suggest that FAM20C plays an important role in hypoxic–ischemic brain injury.

**FIGURE 1:**
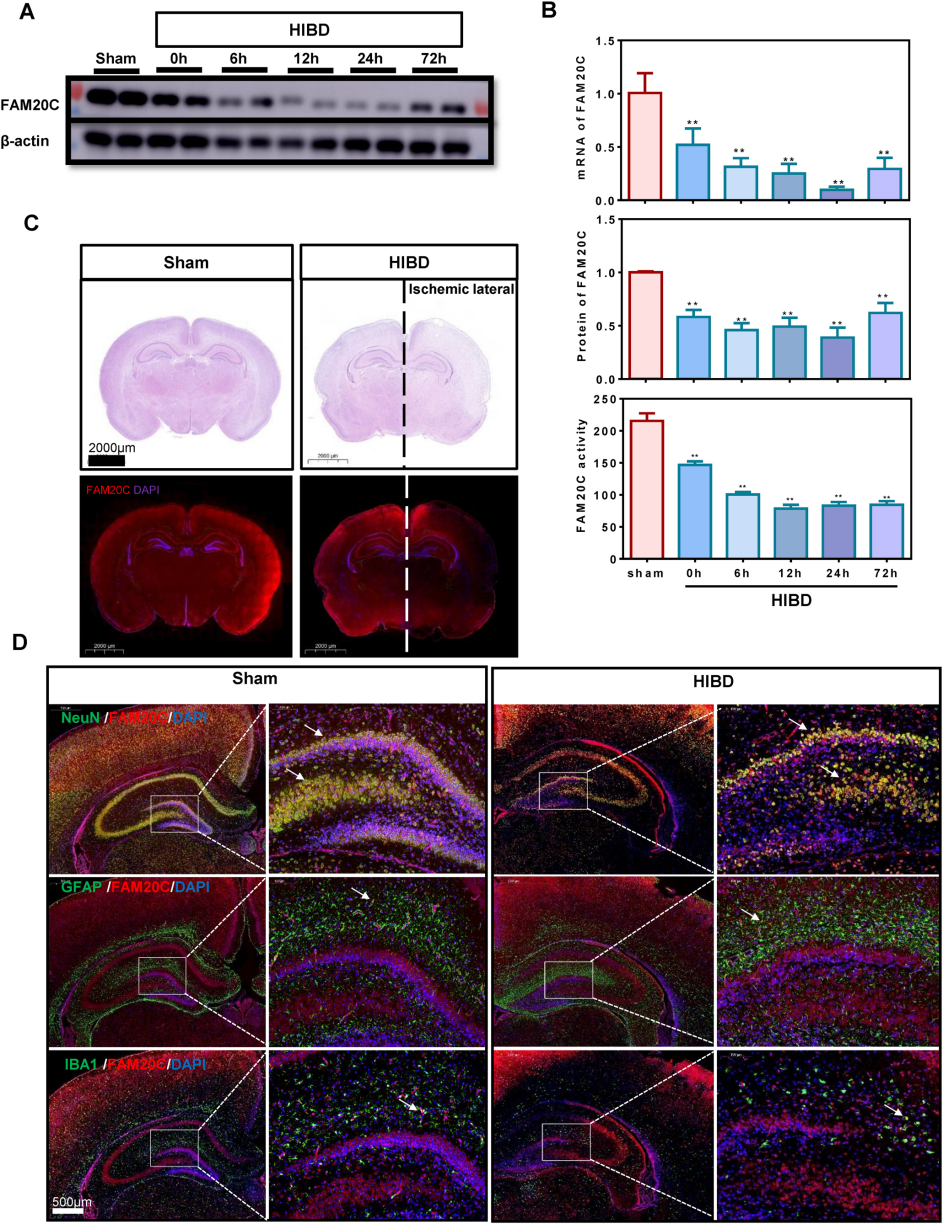
Attenuation of FAM20C in hippocampal neurons after HIBD. (A, B) In the rat hippocampal tissue of the HIBD model, FAM20C's mRNA (B, top), protein expression (B, middle), and kinase activity (B, bottom) were substantially reduced at various time points (0, 6, 12, 24 and 72 h) post‐hypoxia. Notably, the most pronounced downregulation was observed at the 24‐h mark, supported by statistical evidence. Data are reported as mean ± se, from *n* = 3 (B, top), *n* = 4 (B, middle), *n* = 3 (B, bottom) independent biological replicates. One‐way ANOVA *p*‐values are reported. (C) HE staining and immunofluorescence analysis, revealed considerable pathological damage to the brain tissue on the ischemic side 24 h post‐hypoxia (top). Concurrently, a marked decrease in FAM20C protein expression was detected in the ischemic hippocampus and cortex region adjacent to hippocampus (bottom). (D) Specific labeling of glial fibrillary acidic protein (GFAP), neuronal nuclei antigen (NeuN), and ionised calcium‐binding adapter molecule 1 (IBA1) was used to stain astrocytes, neurons and microglia, respectively; nuclei are visualised with DAPI. The arrows represent the co‐localization of GFAP and FAM20, IBA1 and FAM20C, as well as NeuN and FAM20C. Immunofluorescence studies indicated that FAM20C primarily co‐localised with NeuN. A significant reduction in FAM20C‐NeuN co‐localization was observed at 24 h following the ischemic and hypoxic events.

First, the impact of hypoxia‐ischemia on FAM20C was explored. The mRNA and protein expression of FAM20C, and the serine/threonine kinase activity of FAM20C, were detected in the ischemic hippocampal tissue of the HIBD animal model (Figure 1A,B). The results showed that FAM20C is inhibited at 0, 6, 12, 24 and 72 h post‐hypoxia‐ischemia; the most significant inhibition occurs at 24 h. HE staining and immunofluorescence analyses were conducted on coronal sections of the brain 24 h post‐ischemia. The results showed that FAM20C is significantly downregulated in the ischemic area in HIBD, especially in the hippocampus region and the cortex region adjacent to the hippocampus (Figure 1C). FAM20C is mainly located in neurons, and the number of neurons is reduced in ischemic hippocampal tissue (Figure 1D). These findings suggest that hypoxia‐ischemia leads to a reduced number of neurons in the ischemic hippocampal tissue, accompanied by downregulation of FAM20C expression.

### 2.2 Inhibition of FAM20C Mediated by HIBD Leads to Cognitive Impairment by Affecting Neuronal Differentiation

**FIGURE 2:**
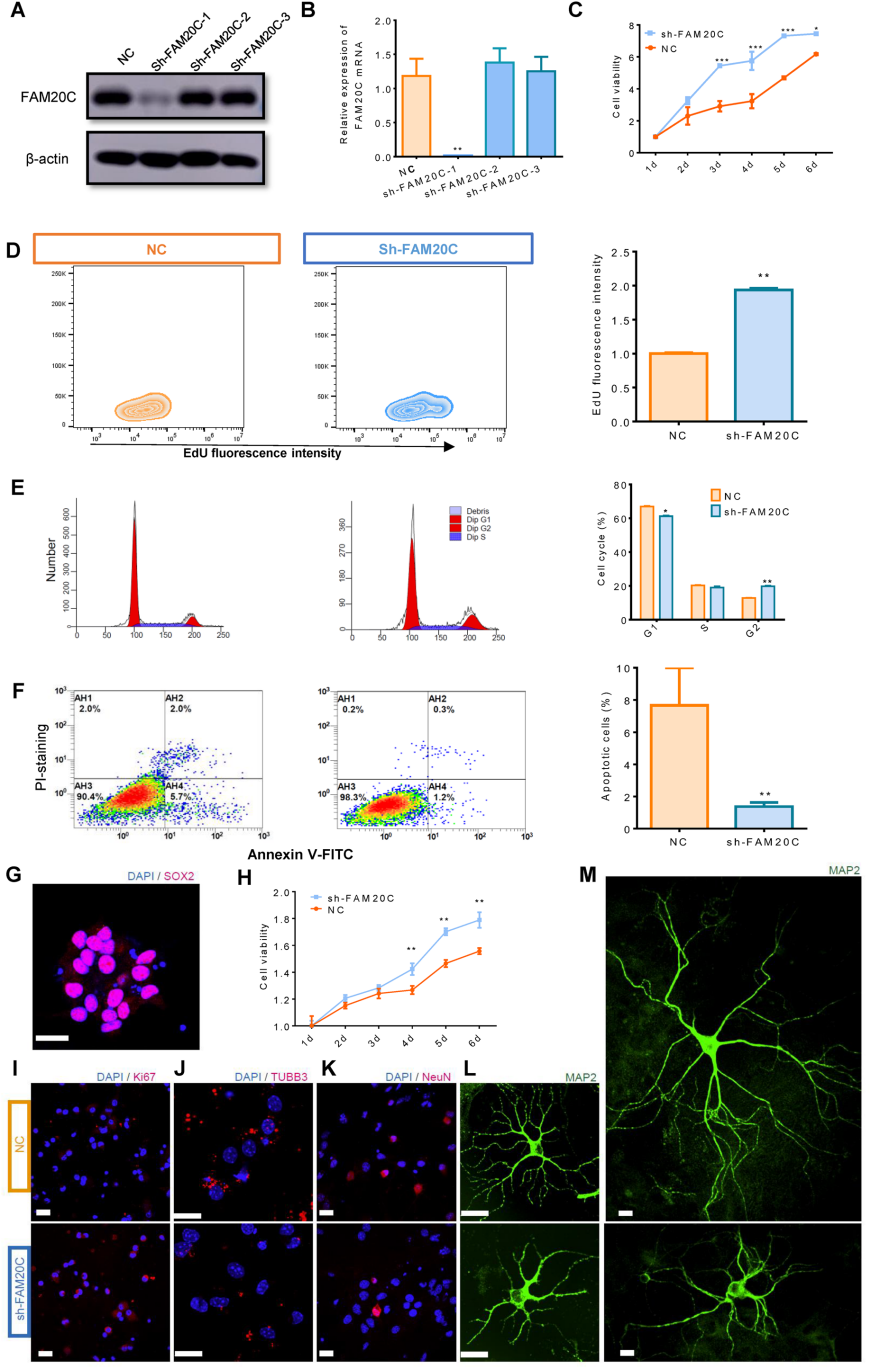
Regulation of PC12 cell proliferation and differentiation by FAM20C. (A, B) Investigation into FAM20C expression demonstrated that a knockdown lentivirus (sh‐FAM20C‐1) significantly reduced FAM20C protein (A) and mRNA levels (B). Lentivirus sh‐FAM20C‐1 was selected for subsequent FAM20C‐knockdown experiments. Data are reported as mean ± se, from *n* = 3 independent biological replicates. One‐way ANOVA *p*‐values are reported. (C) The CCK‐8 assay indicated that FAM20C knockdown substantially enhanced cell viability in PC12. Data are reported as mean ± se, from *n* = 3 independent biological replicates. Two‐way ANOVA *p*‐values are reported. (D) A Significant increase in cell proliferation after FAM20C knockdown was observed, as detected by the fluorescence intensity of EdU through flow cytometry. Data are reported as mean ± se, from *n* = 4 independent biological replicates. Unpaired *t*‐test *p*‐values are reported. (E) A reduction in cell population at the G1 phase of the cell cycle was observed, accompanied by an increase in G2‐phase cells, while the S‐phase population remained unchanged, suggesting dysregulation of cell cycle progression. (F) PI/Annexin V staining and subsequent flow cytometric analysis revealed a decrease in cell apoptosis after FAM20C suppression. Data are reported as mean ± se, from *n* = 3 independent biological replicates. Unpaired *t*‐test *p*‐values are reported. (G) SOX2 labels NSCs. (H) The CCK‐8 assay indicated that FAM20C knockdown substantially enhanced cell viability in NSCs. Data are reported as mean ± se, from *n* = 5 independent biological replicates. Two‐way ANOVA *p*‐values are reported. (I, J) Knockdown of FAM20C results in increased proliferation of NSCs by Ki67 staining(I), decreased expression of the neural differentiation marker TUBB3 at 3 days(J) and NeuN at 5 days (K) post‐induction of differentiation, and impaired neuronal development as indicated by MAP2 staining at 7 days (L) and 14 days (M) post‐induction. Bar = 20 μm.

To explore the role of FAM20C inhibition induced in neuronal cells, we engineered lentiviruses (sh‐FAM20C) to suppress FAM20C expression in the PC12 neuronal cell line. Analysis of protein and mRNA levels revealed a significant downregulation of FAM20C by the sh‐FAM20C‐1 lentivirus (Figure 2A,B). The sh‐FAM20C‐1 lentivirus was used in the following experiments. Suppression of FAM20C induced increases in cell viability (Figure 2C) and proliferation (Figure 2D), alteration of cell cycle (Figure 2E) and the reduction in cell apoptosis (Figure 2F). Further research found that FAM20C has an impact on neuronal differentiation. In cultured neural stem cells (NSCs) (Figure 2G), knockdown of FAM20C inhibits the transition of NSCs into neurons (Figure 2J,K) and impairs neuronal development (Figure 2L,M), while promoting cell proliferation (Figure 2H,I).

**FIGURE 3:**
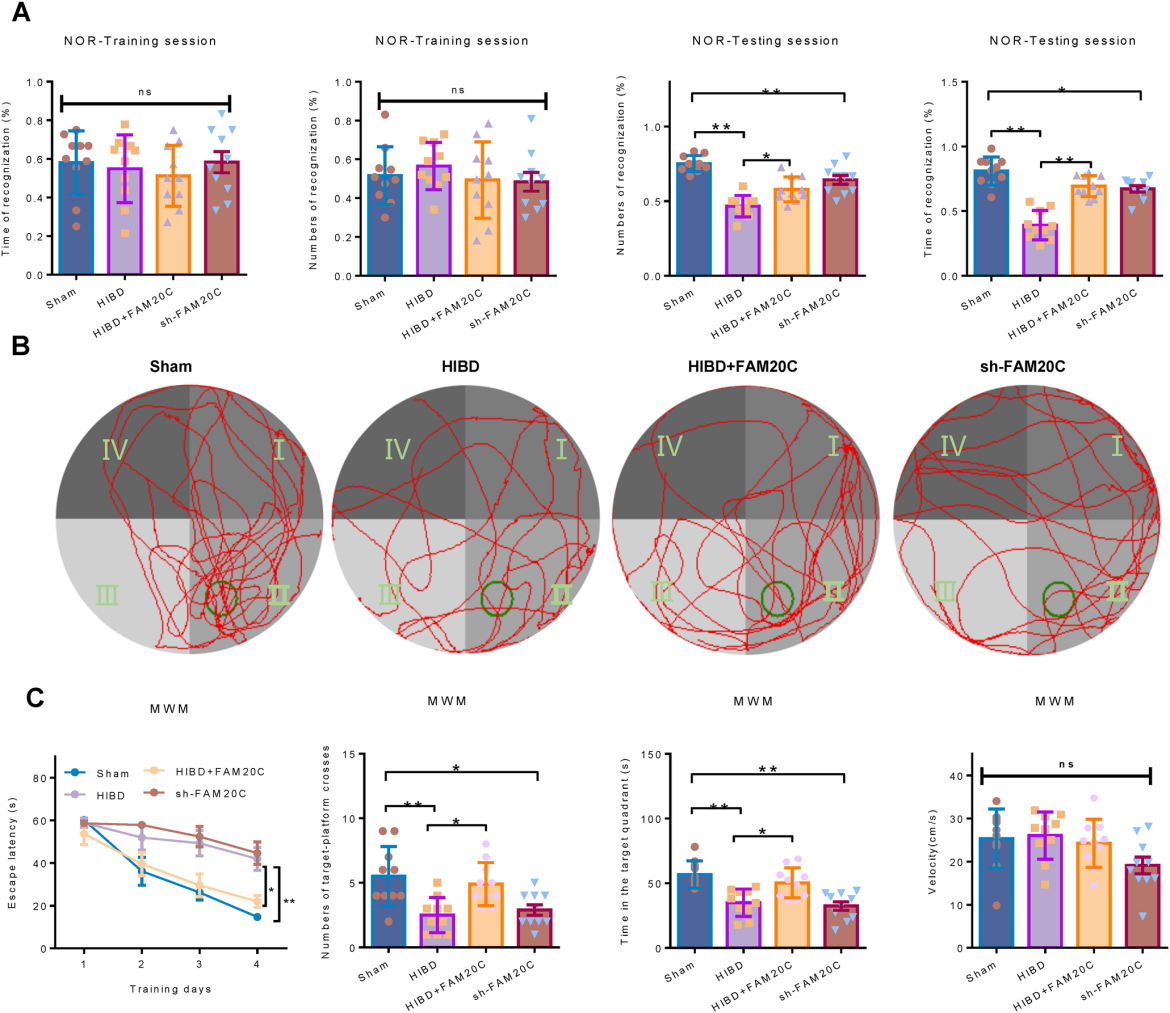
Impact of FAM20C on cognitive impairments induced by HIBD. (A) NOR task: During the initial training phase, object recognition frequency and duration do not differ significantly between the groups (left). Contrastingly, at the test phase, notable disparities were observed in interaction with a novel object between HIBD and Sham groups, sh‐FAM20C and Sham, and HIBD+FAM20C and HIBD groups (right). (B) Tracking plots from MWM: The target platform was located in the second quadrant. (C) In the MWM assessments, significant variations are observed in the escape latency of the four training days (left), platform crossing events, and time spent within the designated target quadrant among the HIBD, sh‐FAM20C, and HIBD+FAM20C groups compared to the Sham group (middle). Uniform swimming speeds across four groups ruled out motor function impairments (right). The groups were set as Sham, HIBD, sh‐FAM20C (knockdown FAM20C), and HIBD+FAM20C (FAM20C overexpressed before HIBD). Data are reported as mean ± se, from *n* = 10 independent biological replicates. One‐way ANOVA *p*‐values are reported.

The hippocampus and cortex are crucial for the execution of cognitive functions [6]. Neuronal differentiation and maturation are critical determinants of cognitive capabilities [7]. In order to ascertain the effect of FAM20C on cognitive functions, we performed behavioural assessments. The Novel Object Recognition (NOR) (Figure 3A) revealed that during the testing phase, the HIBD model group exhibits significantly reduced recognition frequency and duration compared to the Sham group, and overexpression of FAM20C partially reverses this decline. FAM20C knockdown decreases recognition frequency and duration compared to the Sham group. During the training phase, no significant differences were observed among groups. In the Morris Water Maze (MWM) (Figure 3B,C), the HIBD group displayed prolonged escape latency during the training phase, which was alleviated by FAM20C overexpression. During the testing phase, the HIBD group shows significantly fewer platform crossings and reduced target quadrant exploration time, and these deficits are partially rescued by FAM20C overexpression. FAM20C knockdown reduces platform crossings and target quadrant exploration time compared to the Sham group. No significant intergroup differences in swimming speed were detected. Collectively, NOR and MWM data suggest that FAM20C inhibition impairs learning and spatial memory, while its overexpression mitigates HIBD‐induced cognitive deficits. These findings demonstrated that HIBD‐mediated suppression of FAM20C contributes to impaired neuronal differentiation and subsequent cognitive dysfunction.

### 2.3 KRAB‐Associated Protein 1 (KAP1) is a Direct Substrate of FAM20C

**FIGURE 4:**
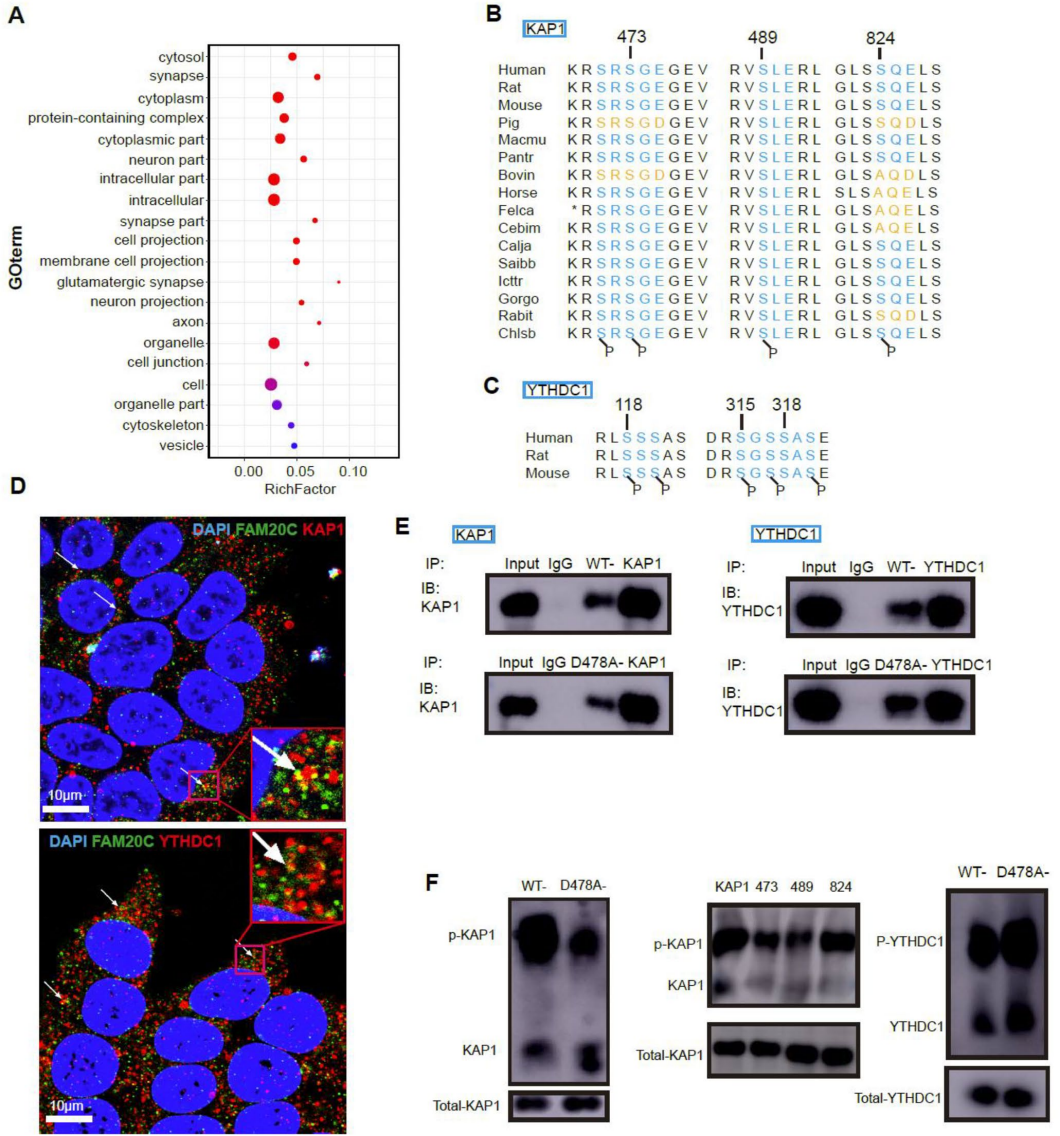
Investigation and validation of FAM20C's direct substrates. (A) Proteomic analysis through gel strip detection following immunoprecipitation with a FAM20C‐specific antibody revealed that most of the proteins that interacted with FAM20C in rat hippocampal tissue were implicated in neuronal composition and functionality, as GO analysis indicated. (B) Bioinformatic analysis using the UniProt database revealed three putative FAM20C‐mediated phosphorylation motifs (Ser‐x‐Glu/pSer) on KAP1 (S473, S489 and S824), supported by homology‐based sequence alignment of evolutionarily conserved residues. (C) YTHDC1 phosphorylation occurred at S118, S315 and S318, and evolutionary conservation analysis results were also presented. (D) Immunofluorescence with specific antibodies demonstrated endogenous protein colocalization with FAM20C and either KAP1 or YTHDC1, indicating molecular interactions. (E) Various plasmids, including wild‐type FAM20C, KAP1 and YTHDC1, along with the kinase‐inactive mutant D478A‐FAM20C, were constructed and co‐transfected into HEK293T cells in specified combinations. Subsequent Co‐IP confirmed protein interactions for both wild‐type FAM20C with KAP1 and the D478A‐FAM20C mutant with KAP1. (F) Phosphorylation changes in KAP1 and YTHDC1 were assessed post‐Co‐IP via phospho‐western blotting (Phos‐WB). KAP1 phosphorylation was reduced by the FAM20C D478A mutation; however, YTHDC1 phosphorylation appeared unaffected. Mutant plasmids S473A‐KAP1, S489A‐KAP1 and S824A‐KAP1 diminished KAP1 phosphorylation when co‐expressed with FAM20C plasmids and analysed via Phos‐WB, particularly following S473A and S489A mutations.

FAM20C, a serine/threonine kinase, mediates its biological effects through the phosphorylation of specific substrates. Prior to this study, the kinase substrate of FAM20C in the nervous system remained unexplored. Based on the FAM20C kinase's motif signature (Ser‐x‐Glu/pSer), mass spectrometry analysis after immunoprecipitation (IP) and UniProt database, which contains protein sequences and phosphorylation sites, were used to find FAM20C substrates in the nervous system, and subsequent verification through molecular biological techniques. Our analysis revealed that proteins interacting with FAM20C in hippocampal tissue relate to neuronal composition and function (Figure 4A), corroborating predictions from computational models described in prior research [5]. Further investigation found six potential substrates of FAM20C: KRAB‐associated protein 1 (KAP1), YTH domain‐containing protein 1 (YTHDC1), adenosine deaminase RNA specific (ADAR), diacylglycerol lipase‐alpha (DAGLA), sequestosome 1 (SQSTM1), and scaffold attachment factor B (SAFB), all preserve conserved potential phosphorylation motifs for FAM20C (Figures 4B,C and S2). We constructed plasmids encoding FAM20C and its catalytically inactive variant D478A‐FAM20C, along with plasmids for the proteins KAP1, YTHDC1, ADAR, DAGLA, SQSTM1 and SAFB, to investigate molecular interactions between KAP1/YTHDC1/ADAR/DAGLA/SQSTM1/SAFB and FAM20C/D478A‐FAM20C, in HEK293T cells. Our findings indicate that, except for SQSTM1, the remaining five proteins bind to both FAM20C and D478A‐FAM20C, but the D478A mutation does not impede the association with these proteins (Figures 4E and S2). Subsequently, Phos‐WB was used to detect phosphorylated bands of these potential substrates. Notably, the D478A mutation results in a marked reduction in phosphorylation of KAP1, phosphorylation of YTHDC1 remains unchanged (Figure 4F), and no effective phosphorylation bands of ADAR, DAGLA and SAFB were detected (data not shown). To further probe potential phosphorylation sites within KAP1, we constructed mutant plasmids of KAP1 (S473A, S489A and S824A). Phosphorylation levels of KAP1 decrease following the S473A and S489A mutations, which identifies these residues as likely phosphorylation sites for FAM20C (Figure 4F). Immunofluorescence assays demonstrated co‐localization of endogenous FAM20C with YTHDC1 and KAP1 in PC12 cells, implying a specific interaction (Figure 4D). Collectively, these data suggest that KAP1 acts as a specific substrate of FAM20C. Although YTHDC1 is not a substrate, it interacts with FAM20C, hinting at a significant role in FAM20C's biological functions.

### 2.4 FAM20C Affects the Establishment ofH3K9me3 Modification on LINE1 DNA by Regulating the Formation of theYTHDC1‐NCL‐KAP1‐LINE1 RNA Complex

**FIGURE 5:**
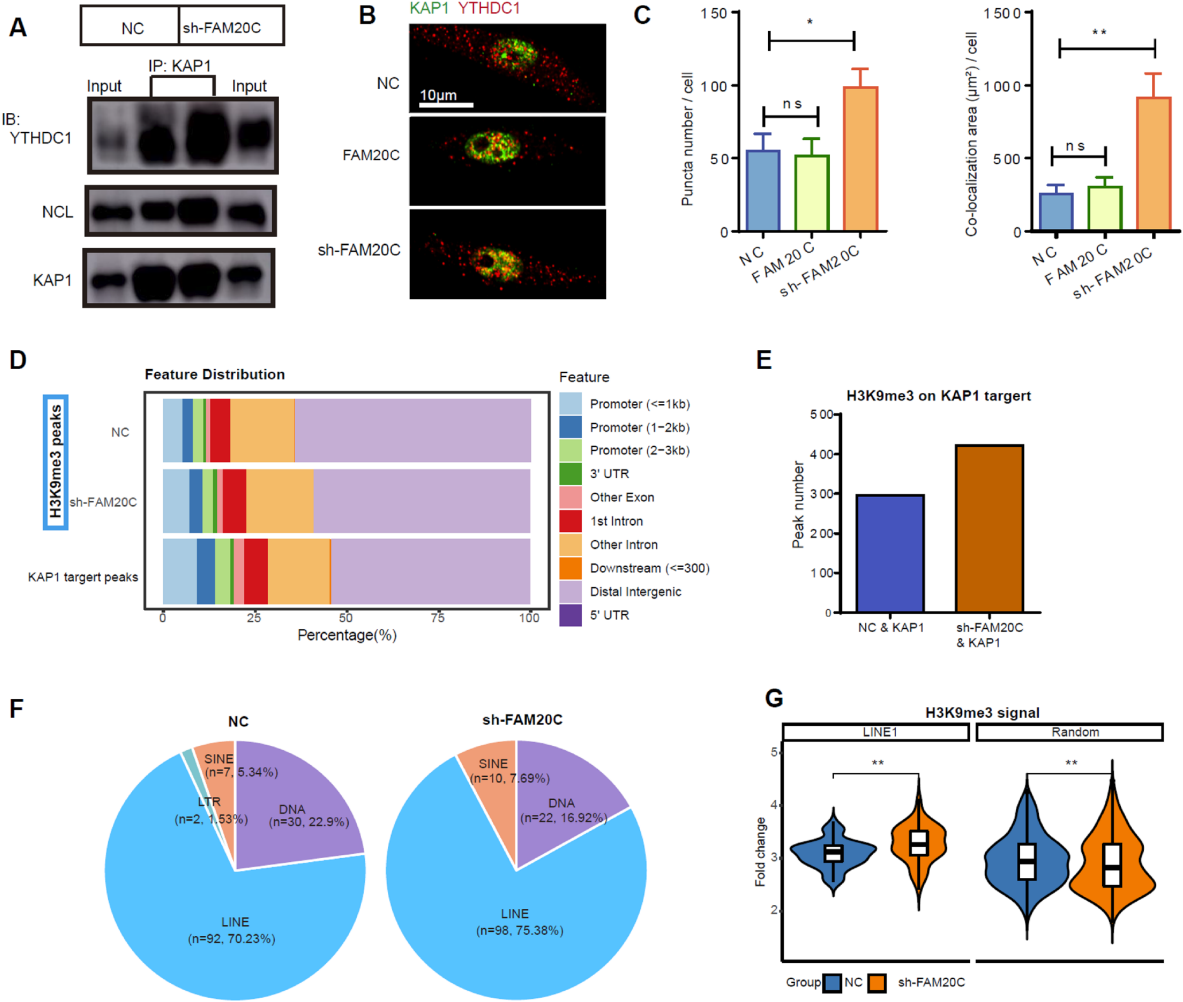
FAM20C is a modulator of the YTHDC1‐NCL‐KAP1 complex formation and influences the establishment of H3K9me3 modification on LINE1 DNA. An increase in the interaction among KAP1, YTHDC1 and NCL in PC12 cells was observed following FAM20C knockdown, by Co‐IP using KAP1 as a reference, and concomitant overexpression of KAP1 and YTHDC1. (B) The association of endogenous YTHDC1 with KAP1 in PC12 cells was visualised through immunofluorescence. (C) Quantitative analysis demonstrated the number and extent of colocalization points within cells were augmented when FAM20C was reduced, and had no change when FAM20C was overexpressed. Data are reported as mean ± se, from *n* = 3 independent biological replicates. Unpaired *t*‐test *p*‐values are reported. (D) ChIP‐seq was used to study the effects of FAM20C on DNA H3K9me3 modification and to detect KAP1 target DNA. The first and second rows of bar charts represent the feature distribution of H3K9me3 modification peaks in the NC group and sh‐FAM20C group, respectively. The third row of the bar chart represents the feature distribution of KAP1 target peaks, which is more similar to the sh‐FAM20C distribution. (E) After comparing sequences using bedtools intersect, the number of H3K9me3 modification peaks on KAP1 targets was counted. Knocking down FAM20C increased the number of H3K9me3 modification peaks on KAP1 targets. (F) Proportional distribution of transposons among H3K9me3 peaks. The proportion of LINE in the sh‐FAM20C group increased. (G) H3K9me3 signals on LINE1 DNA increased, while signals on random DNA slightly decreased. Wilcox test *p*‐values are reported.

Previous studies have reported an interaction between KAP1 and YTHDC1 regulating the formation of the YTHDC1‐NCL‐KAP1‐LINE1 RNA complex, affecting embryonic stem cell differentiation [8]. Our investigations probed the potential influence of FAM20C on the establishment of this complex. Co‐immunoprecipitation (Co‐IP) showed that FAM20C knockdown enhanced the interaction among KAP1, YTHDC1 and NCL, in PC12 cells co‐expressing KAP1 and YTHDC1 (Figure 5A). Immunofluorescence analyses corroborated this finding by showing increased endogenous interactions between KAP1 and YTHDC1 after FAM20C knockdown (Figure 5B,C). Therefore, these data suggest a regulatory role for FAM20C in the formation of the YTHDC1‐NCL‐KAP1‐LINE1 RNA complex.

Furthermore, ChIP‐seq data revealed that FAM20C knockdown correlated with a reduction in H3K9me3 modification in distal intergenic regions (Figure 5D), and an increase in peaks corresponding to KAP1‐mediated H3K9me3 modification (Figure 5E). Additionally, there was a shift in the transposon landscape, with long interspersed nuclear elements (LINE) DNA increasing from 70.23% to 75.38%, short interspersed nuclear elements (SINE) DNA from 5.34% to 7.69%, and long terminal repeat retrotransposons (LTR) DNA from 6.86% to 7.23% (Figure 5F). LINE1 is the dominant and most active subfamily, comprising approximately 80%–90% of all LINE sequences in the genome. Notably, the H3K9me3 signal specifically on LINE1 DNA intensified, whereas it slightly diminished on random DNA (Figure 5G). These ChIP‐seq findings suggest a selective enrichment of KAP1‐driven H3K9me3 modification on LINE1 DNA following the knockdown of FAM20C.

### 2.5 FAM20C Affects Cell Differentiation by Regulating the m6A Modification of LINE1 RNA

**FIGURE 6:**
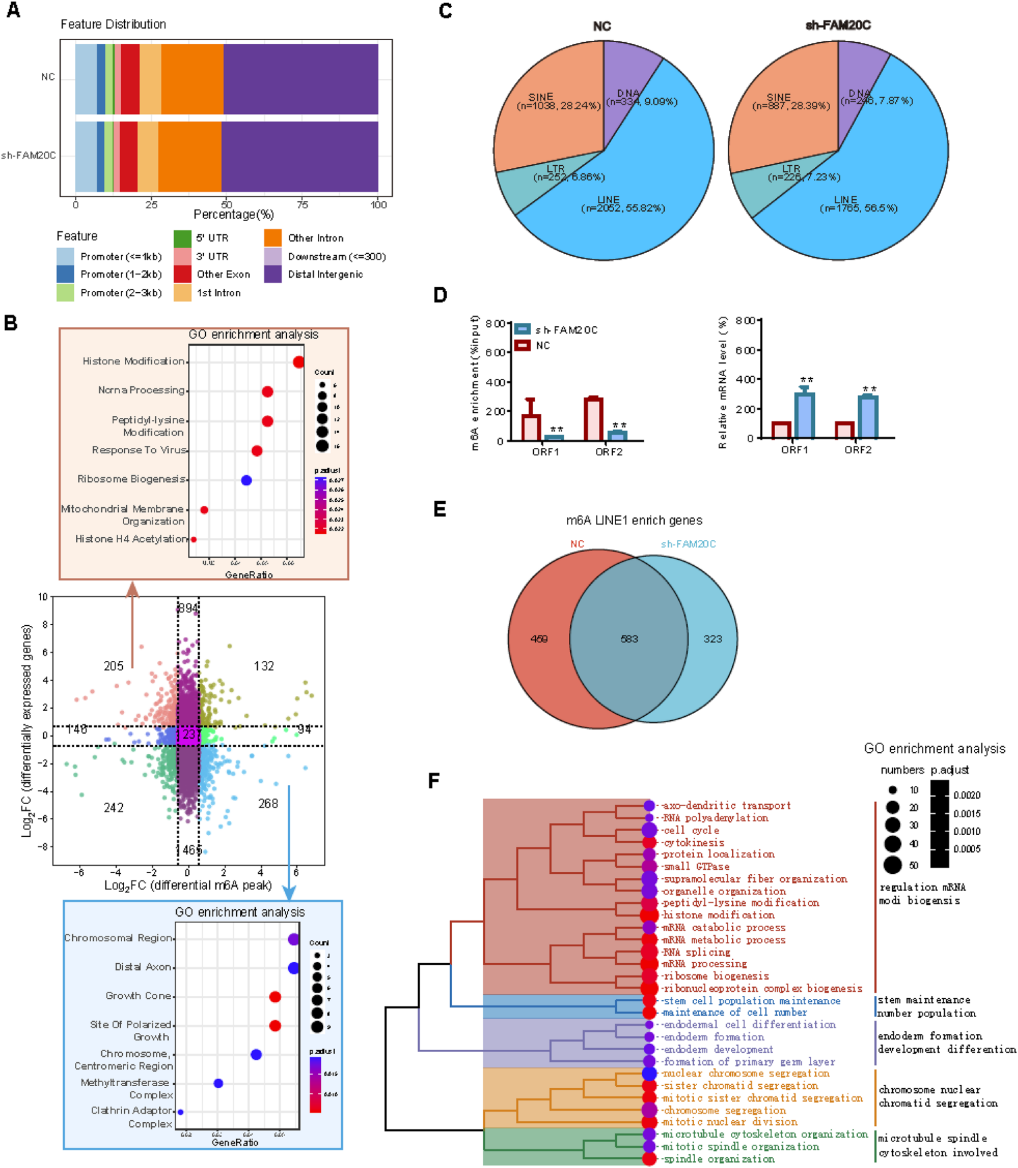
FAM20C influences m6A modification LINE1 RNA‐mediated differentiation. (A) Analysis of m6A‐modified RNA feature distribution indicated that FAM20C knockdown did not markedly alter this distribution. (B) Integration of m6A‐seq and RNA‐seq datasets demonstrated the data distribution and GO enrichment analysis. The upper panel demonstrated the enriched Gene Ontology (GO) terms associated with diminished m6A modification and enhanced transcription levels consequent to FAM20C knockdown. And, the lower panel depicts the enriched GO terms corresponding to augmented m6A modification and reduced transcription levels. (C) The proportional distribution of transposons remains essentially unchanged. (D) m6A‐PCR revealed a diminished m6A signal enrichment on ORF1 and ORF2 (left). Conversely, qRT‐PCR indicated augmented mRNA expression for both ORF1 and ORF2. Data are reported as mean ± se, from *n* = 3 independent biological replicates. Unpaired *t*‐test *p*‐values are reported. (E) A Venn diagram highlighted 323 unique genes linked to m6A‐modified LINE1 RNA, post‐FAM20C suppression. (F) GO enrichment analysis of the 323 unique genes in the sh‐FAM20C cohort.

YTHDC1 operates as a reader of m6A‐modified RNA. Previous research indicates YTHDC1 influences LINE1‐NCL interaction and KAP1 chromatin on recognising LINE1 RNA with m6A modification [8]. Thus, this study explores whether FAM20C correlates with m6A modification in LINE1 RNA. m6A‐seq data demonstrated that FAM20C knockdown did not alter the overall pattern of RNA m6A modification (Figure 6A). Combined with whole‐transcriptome GO enrichment analysis, it was found that when m6A modification is downregulated and its transcription level is upregulated, the biological processes Histone Modification and ncRNA Processing are enriched, suggesting that FAM20C inhibition promotes the establishment of histone modification on non‐coding RNA (Figure 6B), corroborating the findings presented in Figure 5G. In contrast, when m6A modification increases and its transcription levels decrease, the biological processes associated with distal axons are enriched, suggesting that FAM20C suppression impairs axonal functionality, aligning with observations in Figure 2. Although FAM20C suppression did not substantially affect the distribution of transposons (Figure 6C), m6A‐PCR and qRT‐PCR analyses revealed decreased m6A modification on reverse transcription‐related proteins, ORF1 and ORF2, coupled with increased mRNA expression (Figure 6D). Upon activation, the LINE1 element undergoes transcription to produce RNA, which is subsequently translated into two functional proteins: ORF1 serves as an RNA‐binding protein that facilitates the transport of the nucleic acid substrate, while ORF2 possesses dual catalytic activities comprising endonuclease and reverse transcriptase functions. These proteins synergistically mediate the retrotransposition process of the LINE1 element through coordinated enzymatic actions. These results indicate that FAM20C inhibition may enhance LINE1 reverse transcription activity. Finally, GO enrichment analysis of the sh‐FAM20C group‐specific LINE1 genes highlighted the enrichment of biological processes linked to stem cell maintenance, development and differentiation (Figure 6E,F), suggesting that FAM20C affects the m6A modification of LINE1 RNA and mediates neuronal differentiation.

### 2.6 E2F4 Is a Potential Core Transcription Factor for FAM20C in HIBD

**FIGURE 7:**
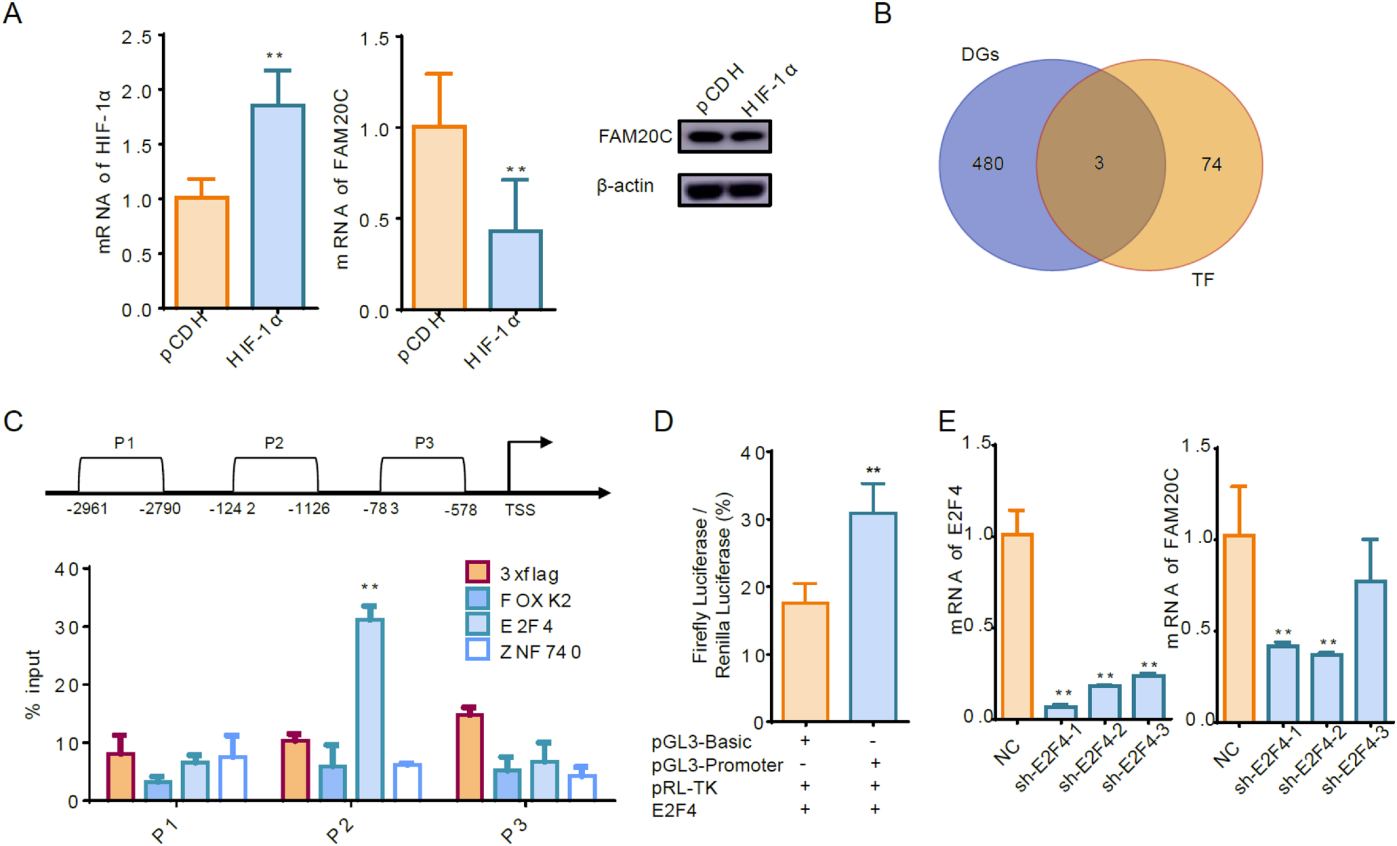
Screening and Identification of core transcription factors regulating FAM20C. (A) After HIF‐1α overexpression in PC12 cells via viral transduction, qRT‐PCR and Western blot analysis demonstrated that HIF‐1α overexpression attenuated FAM20C levels. Data are reported as mean ± se, from *n* = 3 independent biological replicates. Unpaired *t*‐test *p*‐values are reported. (B) The establishment of the HIBD rat model enabled proteomic screening to identify 483 proteins downregulated in the ischemic cerebral hemisphere. Among these, three proteins were also predicted to serve as transcription factors for FAM20C. Data are reported as mean ± se, from *n* = 3 independent biological replicates. (C) After transfection with overexpression constructs for FOXK2, E2F4 and ZNF740, ChIP‐PCR was used to verify their binding affinity to the FAM20C promoter region. Based on promoter sequence data, three primer pairs targeting the FAM20C promoter were designed. Notably, E2F4 exhibited specific binding to the P2 region of this promoter. (D) The promoter region of FAM20C was cloned into the pGL3 dual‐luciferase reporter plasmid. The results further revealed a direct interaction between E2F4 and the promoter region of FAM20C. (E) The knockdown of E2F4 using lentivirus resulted in reduced FAM20C mRNA levels in PC12 cells, as evidenced by qRT‐PCR analysis. Data are reported as mean ± se, from *n* = 3 independent biological replicates. One‐way ANOVA *p*‐values are reported.

To investigate if the core transcription factors regulating the expression of FAM20C in HIBD exist, the transcription factor hypoxia‐inducible factor 1‐alpha (HIF‐1α) was initially considered as a candidate. The subsequent study presented a marked decline in both the transcriptional and protein levels of FAM20C after HIF‐1α overexpression in PC12 cells (Figure 7A), indicating HIF‐1α did not act as a direct transcription factor for FAM20C.

To find transcription factors specifically regulating FAM20C, we employed proteomics coupled with predictive analyses of transcriptional regulators. Three transcription factors, forkhead box K2 (FOXK2), E2F4, and zinc finger protein 740 (ZNF740), exhibited downregulation in ischemic–hypoxic conditions (Figure 7B). Through lentivirus‐mediated overexpression of three transcription factors, we demonstrated that E2F4 directly interacted with the FAM20C promoter region. This interaction was conclusively verified using chromatin immunoprecipitation coupled with polymerase chain reaction (ChIP‐PCR) analysis and further supported by dual‐luciferase reporter assays (Figure 7C,D). Subsequent experiments demonstrated that knockdown of E2F4 led to a decreased expression of FAM20C mRNA (Figure 7E). Taken together, these findings propose E2F4 as a pivotal transcription factor directly involved in the downregulation of FAM20C in HIBD.

## 3 Discussion

**FIGURE 8:**
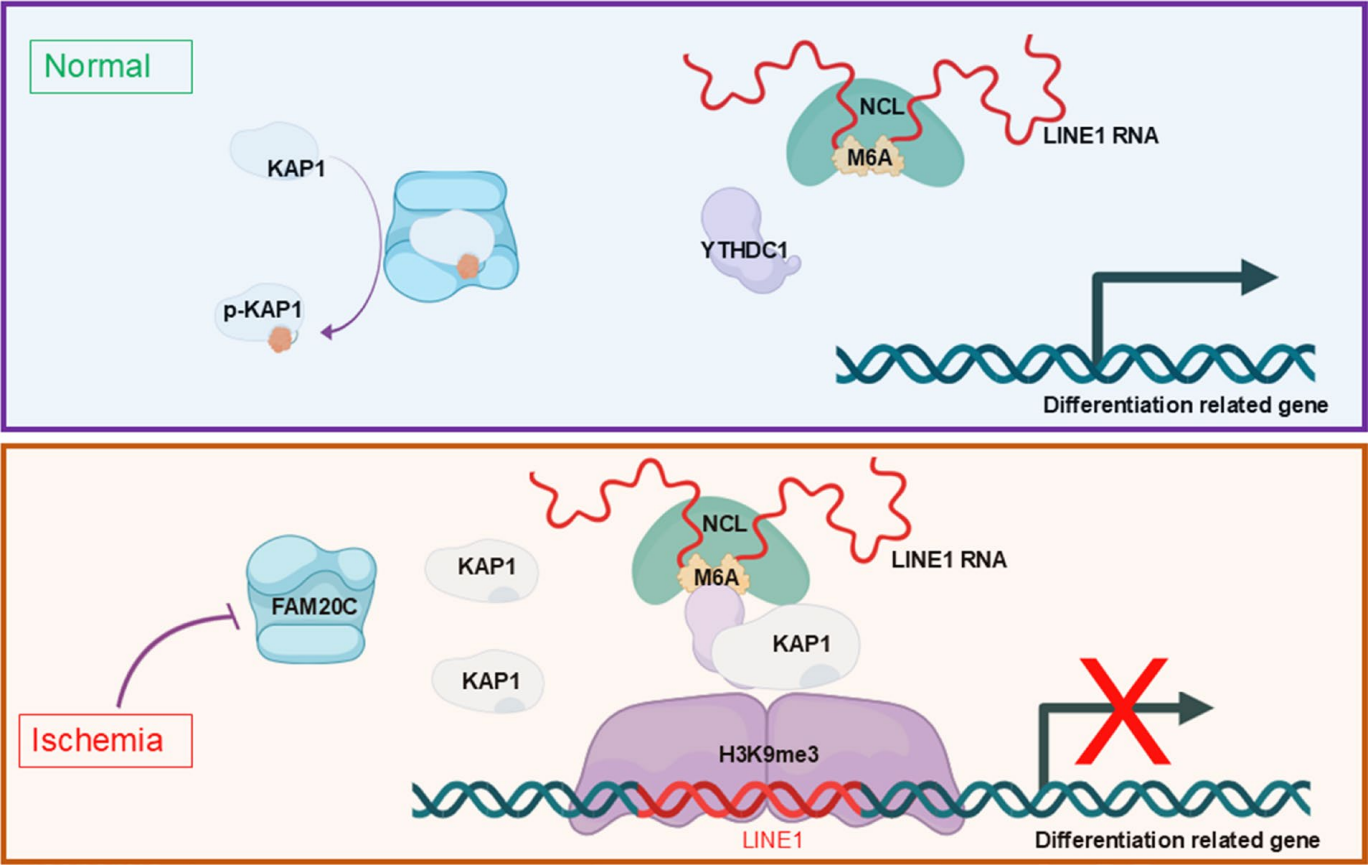
Model showing the role of FAM20C regulating KAP1 and YTHDC1 after HIBD.

This study revealed that HIBD suppresses FAM20C in the hippocampal neurons on the ischemic side, which impairs neuronal differentiation and results in cognitive deficits after HIBD. KAP1 serves as a specific phosphorylation substrate for FAM20C. The regulation of the YTHDC1‐NCL‐KAP1‐LINE1 RNA complex by FAM20C is mediated through modification of KAP1 phosphorylation and LINE1 RNA m6A methylation. These alterations consequently modulate the establishment of the H3K9me3 modification on LINE1 DNA regulating neuronal differentiation (Figure 8). Furthermore, E2F4 is identified as a transcription factor regulated by FAM20C in HIBD. E2F4 binds to the promoter region of FAM20C, and down‐regulates the expression of FAM20C in the process of HIBD.

KAP1 (also known as TRIM28 or TIF1β) is a pivotal transcriptional co‐repressor implicated in a multitude of biological processes, such as DNA repair, cell cycle control, and developmental pathways, which play a significant role in the nervous system. Dysfunction of KAP1 has been linked to disrupted neurogenesis, anxiety‐like behaviour, heightened stress sensitivity, and stress‐related alterations in spatial learning and memory [9]. Phosphorylation modification is a pivotal mechanism for regulating the function of KAP1. The modification of KAP1 at the unique motif regulating its activity and stability affects its interaction with other molecules. Our study displayed the regulation and function of KAP1 phosphorylation modification (S473, S489) in the nervous system. YTHDC1 performs biological functions by recognising m6A‐modified RNA. The m6A RNA prevalence in the brain is linked to the aetiology of neurological disorders and potential oxidative stress [10]. The expression of YTHDC1 is also changed in hypoxic–ischemic conditions, affecting neuronal survival and reparative processes [11]. LINE1, the most abundant retrotransposon in mammalian genomes, makes up over 17% of human and rat DNA. Previous research has associated LINE1 activity with differentiation rates in neurons [12] and explored its role in neonatal hypoxic–ischemic brain damage [13], echoing the findings of our study. Therefore, the molecular mechanism elucidated for FAM20C in our study can find clues in previous studies and is a confirmation and supplement to the previous results.

Hypoxia‐ischemia induces a reduction in neuronal population within ischemic hippocampal tissues, accompanied by a downregulation of FAM20C expression. We postulated that FAM20C inhibition within the nervous system may predominantly exert deleterious effects. Our experimental findings demonstrated that FAM20C suppression leads to diminished cellular apoptosis, enhanced cell viability and proliferation, while simultaneously impairing neuronal differentiation. This phenomenon aligns with previous reports indicating that FAM20C‐mediated phosphorylation regulates odontoblast differentiation [14]. The current investigation revealed that FAM20C inhibition‐induced reduction in apoptosis and promotion of survival/proliferation, coupled with compromised neuronal differentiation, may be mechanistically associated with cell cycle alterations. Experimental data demonstrated that FAM20C knockdown significantly decreased the proportion of cells in G1 phase while increasing that in G2 phase. Neural precursors undergo cell cycle arrest prior to or at least concurrently with neuronal differentiation. Emerging evidence suggests that modulation of G1 phase duration plays a pivotal role in maintaining the balance between progenitor cell maintenance and generation of differentiated neurons [15].

In the hippocampal tissue region, five proteins that interact with FAM20C were identified. Aside from YTHDC1 and KAP1, which are the main focus of our study, there are ADAR, SAFB and DAGLA. ADAR is an enzyme involved in RNA editing and plays an important role in the splicing and editing of various RNA in the nervous system, affecting neural signal transmission and neuronal activity [16]. Under hypoxic–ischemic conditions, the activity of ADAR may change, thereby impacting the RNA editing process, which affects cell survival and nervous system functions [17]. This process relates to the occurrence and development of diseases such as cerebral ischemia and myocardial ischemia. SAFB is an RNA‐binding protein regulating various cellular processes such as transcription, stress response, DNA repair and RNA processing. In the nervous system, SAFB plays a key physiological role by regulating dendritic spine density in hippocampal neurons, affecting synaptic function, inhibiting neural stem cell gliosis and promoting neurogenesis [18]. DAGLA is a critical biosynthetic enzyme in the endocannabinoid system, expressed in neurons and astrocytes throughout the brain, prominently in the cerebellum and hippocampus. DAGLA affects axonal growth and guidance, regulates synaptic release and influences various neurophysiological functions (emotion, cognition, energy balance, pain perception and neuroinflammation) in the nervous system [19]. Elucidating the interaction mechanism between FAM20C and ADAR, SAFB and DAGLA holds crucial implications for understanding both the physiological roles of FAM20C in the nervous system and its pathological contributions to neurological disorders. However, the identification of ADAR, SAFB and DAGLA as specific phosphorylation substrates of FAM20C remains inconclusive due to technical limitations in current Phos‐WB. The compromised detection arises from two primary factors: interference by nonspecific banding patterns obscuring phospho‐signal interpretation and insufficient sensitivity of Phos‐WB to reliably detect low‐abundance phosphorylation events. To overcome these methodological constraints, we propose implementing an advanced phosphoproteomics approach coupled with immunoprecipitation in subsequent investigations.

FAM20C is a serine/threonine protein kinase that participates in various biological functions through the phosphorylation modification of secreted proteins. Following the rationale that three FAM20C substrates (CP, APOE, AFP) were identified as potential HIE biomarkers based on their progressively increasing/decreasing expression patterns across four clinical subgroups (control, mild HIE, moderate HIE, severe HIE), which could enhance their diagnostic utility for HIBD‐induced neurodysplasia, FAM20C is consequently proposed as a putative therapeutic target for neurodevelopmental disorders associated with HIBD. APOE is the primary cholesterol carrier in the nervous system, influencing various neural cellular processes including neuronal growth, membrane repair and remodelling, synaptogenesis, amyloid β‐protein clearance and degradation and neuroinflammation, and is a biomarker for late‐onset Alzheimer's disease [20]. AFP is a biomarker for neurodegenerative diseases (like cerebellar ataxia) [21], and is considered to potentially promote repair after neural injury by regulating neurogenesis‐related signalling pathways [22]. CP is the most important copper carrier in plasma, and its effects are indispensable for the distribution, transport, and storage of copper ions in the body. CP deficiency affects neuronal projection extension, synaptic signal transmission, and cell mRNA translation and is closely related to the occurrence of neurodegenerative diseases [23]. While screening for potential gel substrates of FAM20C through gel protein mass spectrometry, APOE, CP, and AFP were not detected. We believe this result is related to the protein abundance in the hippocampal tissue and the detection accuracy of mass spectrometry; these three proteins might also serve as modification targets for FAM20C to perform important biological functions, which requires further investigation.

HIF‐1α was initially posited as the key transcription factor of FAM20C. Typically, HIF‐1α binds to the promoters of target genes, stimulating their transcription under hypoxic conditions. The exploration results presented a marked decline in both the transcriptional and protein levels of FAM20C after HIF‐1α overexpression, indicating that rather than acting as a direct transcription factor of FAM20C, HIF‐1α may play an ancillary role in its downregulation. Furthermore, E2F4 is identified as a transcription factor regulated by FAM20C in HIBD through proteomic prediction combined with molecular biological validation. E2F4 belongs to the E2F family of transcription factors, which is principally recognised for the regulation of the cell cycle and significant contributions to neuronal development and differentiation. The decrease in E2F4 expression during hypoxic conditions correlates with neuronal survival following ischemic events, as reported [10], aligning with the results obtained in our study.

In conclusion, our research elucidated a novel association between FAM20C and HIBD, laying the groundwork for innovative diagnostic and therapeutic strategies to combat neurodevelopmental disruptions stemming from neonatal HIE. Moreover, utilising both proteomics and molecular biology techniques, this study was the first to screen and identify phosphorylated targets of FAM20C in rat brain tissue. The search for interactive proteins and phosphorylation targets linked to FAM20C in the nervous system paved the way for a deeper understanding of the pathophysiological underpinnings of neurological disorders associated with FAM20C, including treatment‐resistant depression [24], Raine syndrome, and familial cerebral small vessel disease [25]. This study demonstrated clinical translational potential. While AAV vector‐mediated delivery of the FAM20C gene may compensate for HIBD‐induced FAM20C deficiency, potential immune responses to viral vectors or exogenous genes require thorough investigation.

## 4 Materials and Methods

### 4.1 Animal Experiment

The feeding and experimental protocols for the rats adhered to national ethical standards and received approval from the Ethics Committee of Soochow University (approval code SUDA20200824A02). The rodents were acquired from Qizhen Experimental Animal Technology Co. Ltd., Hangzhou, and maintained in an environment with regulated temperature and humidity. The study did not consider sex.

#### 4.1.1 Animal HIBD Model

The model was established on postnatal day seven, employing methodologies described previously [26].

#### 4.1.2 Lentivirus Injection

Within the first 24 h post‐birth, lentiviruses were administered bilaterally into the lateral ventricles of the neonates at coordinates 0.5 mm anterior to the bregma, 1 mm lateral, and 3.5 mm deep, with 3 μL of solution per injection site and an infusion rate of 0.5 μL/min.

#### 4.1.3 Behavioural Assessment

NOR and MWM were conducted at the age of 2 months, following previously established procedures and statistical analyses available [27].

### 4.2 Cell Experiment

Cell lines PC12 (Procell, CL‐0480) and HEK293T (Procell, CL‐0005), both of which were cultured following the guidelines outlined in the manufacturer's operational manual. The PC12 cells were employed to investigate alterations in cell viability, proliferation, apoptosis and differentiation. The HEK293T cells were utilised for probing molecular interactions. The isolation and culture of primary NSCs were performed as described previously [28]. These NSCs were utilised to investigate cellular differentiation.

#### 4.2.1 Lentivirus Infection

PC12 cells were seeded in six‐well plates at a density of 5000 cells per well. Each well was supplemented with 10 μL of lentivirus (integration units ≥ 1 × 10^8^ TU/mL) and 10 μL of transfection reagent. The culture medium was replaced at the 24‐h post‐infection mark. After a 48‐h interval, the cells underwent selection with 1 μg/mL puromycin dihydrochloride (Beyotime, ST551) for an additional 48 h; then, surviving cells were harvested for further analysis.

#### 4.2.2 Cell Viability Assay

The viability of PC12 cells was assessed using CCK‐8 (MCE, HY‐K0301). Cells were plated at 1000 cells per well in 96‐well plates. After adding 10 μL of CCK‐8 solution per well, the cells were incubated at 37°C for 1 h. The absorbance at 450 nm was measured, and readings were taken consecutively for 6 days to monitor viability over time.

#### 4.2.3 Proliferation Assay

The proliferation of cells was quantified via the fluorescence intensity of 5‐ethynyl‐2′‐deoxyuridine (EdU, Beyotime, C0081S) using flow cytometry. Cells were cultured in six‐well plates to achieve 30% confluence and were incubated with a 10 μM solution of EdU for 24 h. Post incubation, cells were collected, prepared according to the manufacturer's protocol and analysed.

#### 4.2.4 Apoptosis Detection

Apoptotic cells were stained using the Annexin V/PI staining kit (BD, 556547) and detection was performed using flow cytometry. Cell treatment and subsequent detection followed the manual's instructions.

### 4.3 Western Blot, Phos‐Tag Western Blot (Phos‐WB), and co‐IP

Western blotting and Co‐IP were conducted following previously established protocols [29]. We utilised several antibodies at the specified dilutions for western blot analysis: rabbit anti‐FAM20C (Abcam, ab154740, 1:1000), rabbit anti‐NCL (Beyotime, AF7548, 1:1000), rabbit anti‐HA (CST, 37240S, 1:2000), mouse anti‐MYC‐tag (Beyotime, AF003S, 1:1000), and rabbit anti‐β‐Actin (CST, 4970S, 1:1000). For Co‐IP, the following reagents were employed: rabbit anti‐FAM20C (Sigma, AV49490), mouse anti‐Flag magnetic beads (Selleck, B26101), mouse anti‐HA magnetic beads (Selleck, B26201), Protein A/G magnetic beads (Selleck, B23201), normal mouse IgG (Santa Cruz Biotechnology, sc‐2025), and normal rabbit IgG (CST, 2729S).

Phos‐WB is an advanced technique that enables concurrent analysis of phosphorylated proteins and their non‐phosphorylated counterparts. The method parallels traditional SDS‐PAGE western blotting with notable distinctions: in Phos‐WB, the separation gel was supplemented with 50 μM Phosbind Acrylamide and 100 μM MnCl_2_ (APExBIO, F4002). Post‐electrophoresis, the gel was immersed in a transfer solution containing 10 mM ethylenediaminetetraacetic acid (EDTA) for 30 s to chelate and remove manganese ions (Mn^2+^). To enhance transfer efficiency, Western Rapid Transfer Buffer (Beyotime, p0572) was employed, facilitating the process at 400 mA for 2 h.

### 4.4 Immunofluorescence

The experimental methodology followed the previously established protocols [30]. Coronal sections of brain tissue, prepared from paraffin‐embedded samples 24 h post‐ischemia–reperfusion, were utilised for immunofluorescence studies. Before analysis, cells designated for immunofluorescence were infected with a lentivirus and subsequently plated onto confocal dishes pre‐coated with 0.1 mg/mL poly‐D‐lysine (Beyotime, ST508), ensuring a seeding density of 10,000 cells per dish.

The following primary antibodies were employed for immunolabelling: rabbit anti‐FAM20C antibody (Abcam, ab154740, 1:200), mouse anti‐KAP1 antibody (Proteintech, 66,630‐1‐Ig, 1:200), mouse anti‐YTHDC1 antibody (Proteintech, 4392‐1‐AP, 1:200), rabbit anti‐YTHDC1 antibody (CST, 77422S, 1:200), mouse anti‐NeuN antibody (Servicebio, GB15138, 1:200), mouse anti‐GFAP antibody (Servicebio, GB12100, 1:200), mouse anti‐IBA1 antibody (Servicebio, GB15105, 1:200), rabbit anti‐MAP2 antibody (CST, 4542S, 1:400), rabbit anti‐SOX2 antibody (Bio‐swamp, PAB30713, 1:400), rabbit anti‐Ki67 antibody (Bio‐swamp, PAB30684, 1:400).

### 4.5 ELISA and Kinase Assays

ELISA was conducted as per the provided protocol by the ELISA kit manufacturer. The specimens were serum samples from experimental rats collected 24 h following ischemia–reperfusion via orbital blood sampling. The ELISA kits employed included those for AFP (Bioswamp, RA20963), APOE (Bioswamp, RA20253), and CP (Bioswamp, RA21014).

The kinase assays were performed in a buffer containing 50 mM imidazole, pH 7.0, 5 mM MnCl_2_, 5 mM MgCl_2_, 25 μM ATP, β‐casein (10 μM) [31], and detected by Kinase Assay Kit (Beyotime, S0155S). Samples consisted of brain tissue lysates containing casein kinases, casein kinase 1/cell kinase 1 (CK1) and casein kinase 2/cell kinase 2 (CK2). Following prior research [32], FAM20C is not sensitive to 200 μM staurosporine (Beyotime, S1883), and the kinase activities of CK1 and CK2 can be effectively inhibited. Therefore, to ensure accurate measurement of FAM20C kinase activity, samples were pre‐treated with 200 μM staurosporine and incubated at 37°C for 30s prior to the assay. Additionally, protein concentrations were quantified using the BCA method (Beyotime, P0010S).

### 4.6 Plasmids and Viruses

The plasmids were engineered by General Biol, based in China. The details of the constructs, including transcript IDs, vectors, and cloning sites, are described as follows: SAFB‐HA (NM_001201338.2, pLVX‐EF1a‐IRES‐Neo, EcoRI‐BamHI); KAP1‐HA, KAP1‐S473A‐HA, KAP1‐S489A‐HA, KAP1‐S824A‐HA (NM_005762.3, pLVX‐EF1a‐IRES‐Neo, EcoRI‐NotI); YTHDC1‐HA, YTHDC1‐MYC (NM_001031732.4, pLVX‐EF1a‐IRES‐Neo, EcoRI‐NotI); DAGLA‐HA (NM_006133.3, pLVX‐EF1a‐IRES‐Neo, EcoRI‐NotI); ADAR (NM_001025107.3, pLVX‐EF1a‐IRES‐Neo, EcoRI‐NotI); SQSTM1‐HA (NM_001142298.2, pLVX‐EF1a‐IRES‐Neo, EcoRI‐NotI); E2F4‐FLAG (NM_001950.4, pLVX‐EF1a‐IRES‐Puro, EcoRI‐XbaI); ZNF740‐FLAG(NM_001004304.4, pLVX‐EF1a‐IRES‐Neo, EcoRI‐NotI); FOXK2‐FLAG (NM_004514.4, pLVX‐EF1a‐IRES‐Puro, EcoRI‐XbaI); FAM20C‐FLAG, FAM20C‐D478A‐FLAG(NM_020223.4, pCDH‐CMV‐MCS‐EF1, EcoRI‐BamHI); HIF‐1α (NM_001530.4, pCDH‐CMV‐MCS‐EF1‐copGFP‐T2A‐Puro, EcoRI‐BamHI).

Lentiviruses for FAM20C knockdown and overexpression were both constructed and extracted by the company (OBIO, China). The information of the sh‐FAM20C lentivirus is as follows: NM_001012238.1, pCLenti‐U6‐shRNA‐CMV‐Puro‐WPRE, 1#, GCTCTTAGCCAAGTTGTTTGA, 2#, GCTATGGAGAACCTTCTTTGT, 3# GCATCACTACGAGACCTTTGA. The overexpression lentivirus FAM20C is: NM_020223.4, GL117 pSLenti‐EF1‐Puro‐CMV‐MCS‐WPRE, EcoRI‐BamHI. The overexpression lentiviruses FOXK2, E2F4, ZNF740, HIF‐1α and KAP1 were packaged by using the PAX2 and PMD2g two‐plasmid system. The experimental methods are as previously described [33].

### 4.7 Dual‐Luciferase Reporter Gene Assay

The dual‐luciferase reporter gene system employed the pGL3 vector, into which the promoter region of FAM20C was cloned to construct the pGL3‐Promoter plasmid. Plasmid construction was performed by a company (Qingke, China). The internal control plasmid pRL‐TK was used. The 48 h prior to harvesting, HEK293T cells were co‐transfected with the aforementioned plasmids and the E2F4‐FLAG overexpression plasmid using Lipo293 Plus Transfection Reagent (Beyotime, C0522). Firefly luciferase and Renilla luciferase were quantified using the Dual‐Luciferase Reporter Assay System detection kit (Promega, E1910), with all experimental procedures strictly adhering to the manufacturer's protocols.

### 4.8 ChIP‐Seq and m6A‐Seq Protocols

The DNA extraction for ChIP‐seq followed the established protocol as reported [33]. The sequencing and preliminary analysis were conducted by the company (BGI, China). For m6A‐seq, the extraction of samples, preparation of sequencing libraries, and initial processing, which included data cleaning and filtering, were performed by the company (SHBIO, China). The reference genome used was mRatBN7.2 (GCA_015227675.2). Transposon analysis was carried out utilising the extensive de novo TE annotator (EDTA) [34] to create an exhaustive library of rat transposons. This compilation was augmented by integrating information from the Repbase and SINE base and employing RepeatModeler. BEDTools intersect was used to calculate transposon‐enriched peaks and H3K9me3 peaks on KAP1 target sites. GO analysis was completed with Hiplot Pro (https://hiplot.com.cn/).

### 4.9 PCR

The RNA extraction and qPCR experimental methods for quantitative real‐time PCR (qRT‐PCR) were performed as previously described [33]. ChIP‐PCR and m6A‐PCR were conducted on DNA isolated via ChIP and on DNA samples prepared for m6A‐seq libraries, respectively, to facilitate quantitative PCR (qPCR) analysis. The primer sequences used in these assays are detailed below:

ORF1, F: 5’‐GAACCCAAGCAACAGAAACCA‐3’, R: 5’‐CCATGTTTGTTTGGCGGGA‐3’;

ORF2, F: 5’‐TCTATGCCCCAAATACAAG‐3’, R: 5’‐AGTTTTCCTCTTAGCACAGC‐3’;

β‐Actin, F: 5’‐AGCCTTAGCCTGGACCCATA‐3’, R: 5’‐CGGACTCATCGTACTCCTGC‐3′；

FAM20C‐P1, F: 5’‐TGCTTACACGCCCAATGAGT‐3’, R: 5’‐CCAGATTTGGACGTCGTGGA‐3’;

FAM20C‐P2, F: 5’‐AGGGTGCGATGAATGCAGAA‐3′, R: 5’‐TGGGCAGCATAGCATAGTGG‐3′；

FAM20C‐P3, F: 5’‐CTCCTGTTCCCAGATGCCTG‐3’, R: 5’‐CCCAGAGGGTCACACACAAG‐3’;

FAM20C, F: 5’‐GCTTGCGCTTTGCACATTG‐3’, R: 5’‐GAGGTTAGGTTAGAGGCTGGA‐3’;

E2F4, F: 5’‐CTCACCACCAAGTTCGTGTC‐3’, R: 5’‐TCTCGATCAGACCGATGCCTT‐3’;

HIF‐1α, F: 5’‐GTCCCAGCTACGAAGTTACAGC‐3’, R: 5’‐CAGTGCAGGATACACAAGGTTT‐3’.

### 4.10 Proteomics

The high‐throughput iTRAQ proteomics data for neonatal asphyxia are from published literature [1]. Weighted gene co‐expression network analysis and principal component analysis were used to identify differential proteins in plasma. IP and TMT mass spectrometry facilitated the detection of proteins that specifically interact with FAM20C in the hippocampal tissues of healthy 7‐day‐old rats, employing immunoprecipitation‐grade anti‐FAM20C antibodies analogous to those used in Western blotting. Subsequently, tandem mass tag (TMT) labeling proteomics was applied to discern differentially expressed proteins in the cerebral tissues following HIBD, with a focus on the ischemic hemispheres 24 h post‐ischemia–reperfusion. Proteomics was conducted by a company (BIOTREE, China).

### 4.11 Homology Sequence Alignment

The UniProt database archives the full‐length amino acid sequences as well as the phosphorylation sites of the proteins. For homologous sequence alignment among diverse species, DNAMAN software was deployed using default parameters.

### 4.12 Statistical Analysis

Statistical analysis was performed using GraphPad Prism. “ns” stands for not significant, and statistical significance was considered at *p* < 0.05 (*p < 0.05, **p < 0.01, ***p < 0.001).

## Author Contributions

X.F. conceived and designed the study. M.L. and L.X. supervised the project. C.F., M.W., and G.L. performed experiments and drafted the manuscript. S.C., D.W., and X.H. conducted animal studies. All authors critically revised the manuscript.

## Ethics Statement

The feeding and experimental protocols for the rats adhered to national ethical standards and received approval from the Ethics Committee of Soochow University (approval code SUDA20200824A02).

## Consent

All authors read and approved the final manuscript.

## Conflicts of Interest

The authors declare no conflicts of interest.

## Supporting information


**Figure S1.** Altered levels of CP, APOE and AFP in HIE patients and HIBD model rats. (A) Weighted gene co‐expression network analysis and principal component analysis implicate CP, APOE, PTK2B and AFP as prospective biomarkers. (B) Utilising a HIBD rat model, ELISA assays conducted after 24 h of hypoxia reveal significant fluctuations in serum CP, APOE and AFP levels. The hypoxic time for Moderate HIBD and Severe HIBD are 1.5 and 2.5 h, respectively. Data are reported as mean ± SE, from *n* = 4 independent biological replicates. One‐way ANOVA *p*‐values are reported.
**Figure S2.** Confirmation of Proteins Interacting with FAM20C. After gel strip proteomics, ADAR, DAGLA, SQSTM1 and SAFB emerge as potential FAM20C interfactors. Subsequent homology analysis and validation of protein interactions were undertaken. (A) ADAR possess three putative phosphorylation sites (S629, S636, S823). Co‐transfection of ADAR with either wild‐type FAM20C or the D478A‐FAM20C mutant variant into HEK293T cells followed by Co‐IP confirms their interaction. (B) DAGLA, with phosphorylation sites at S727, S732 and S806, similarly interacts with FAM20C upon co‐transfection and Co‐IP assay in HEK293T cells. (C) SQSTM1, featuring a single phosphorylation site at S272, does not exhibit interaction with FAM20C. (D) SAFB, bearing three phosphorylation sites analogous to those in ADAR, is confirmed to interact with FAM20C through Co‐IP assays upon co‐transfection in HEK293T cells.

## Data Availability

ChIP‐seq, m6A‐seq and mass spectrometry data have been saved in China National Center for Bioinformation (CNCB, PRJCA030767).
