# Supplementary figures and images for: FAM20C Modulates Neuronal Differentiation in Hypoxic–Ischemic Brain Damage via KAP1 Phosphorylation and LINE1 RNA m6A‐Dependent H3K9me3 Regulation
Source: Cell Prolif. 2025 Jun 13;59(1):e70073. doi: 10.1111/cpr.70073 (PMC12774626; doi:10.1111/cpr.70073)

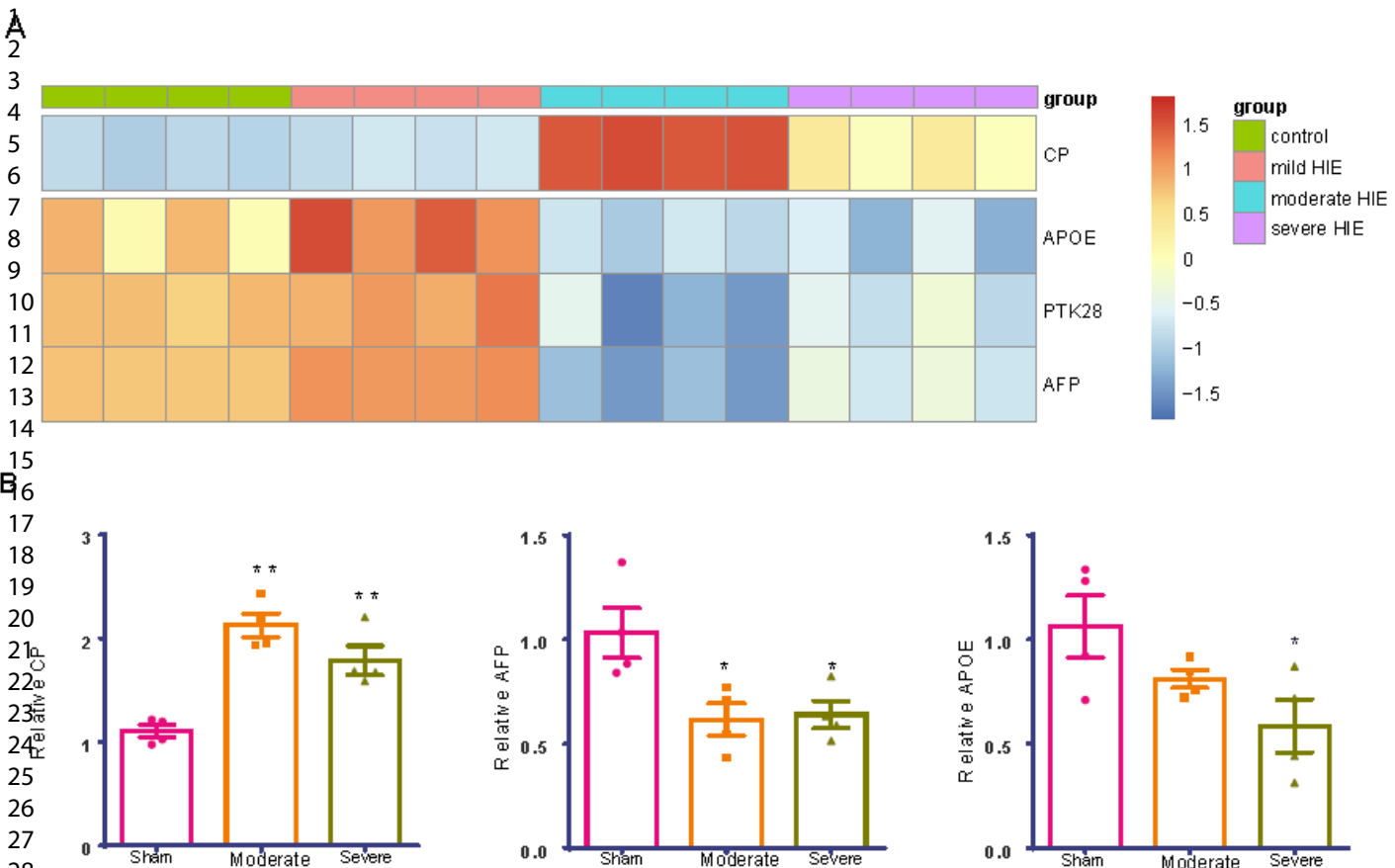

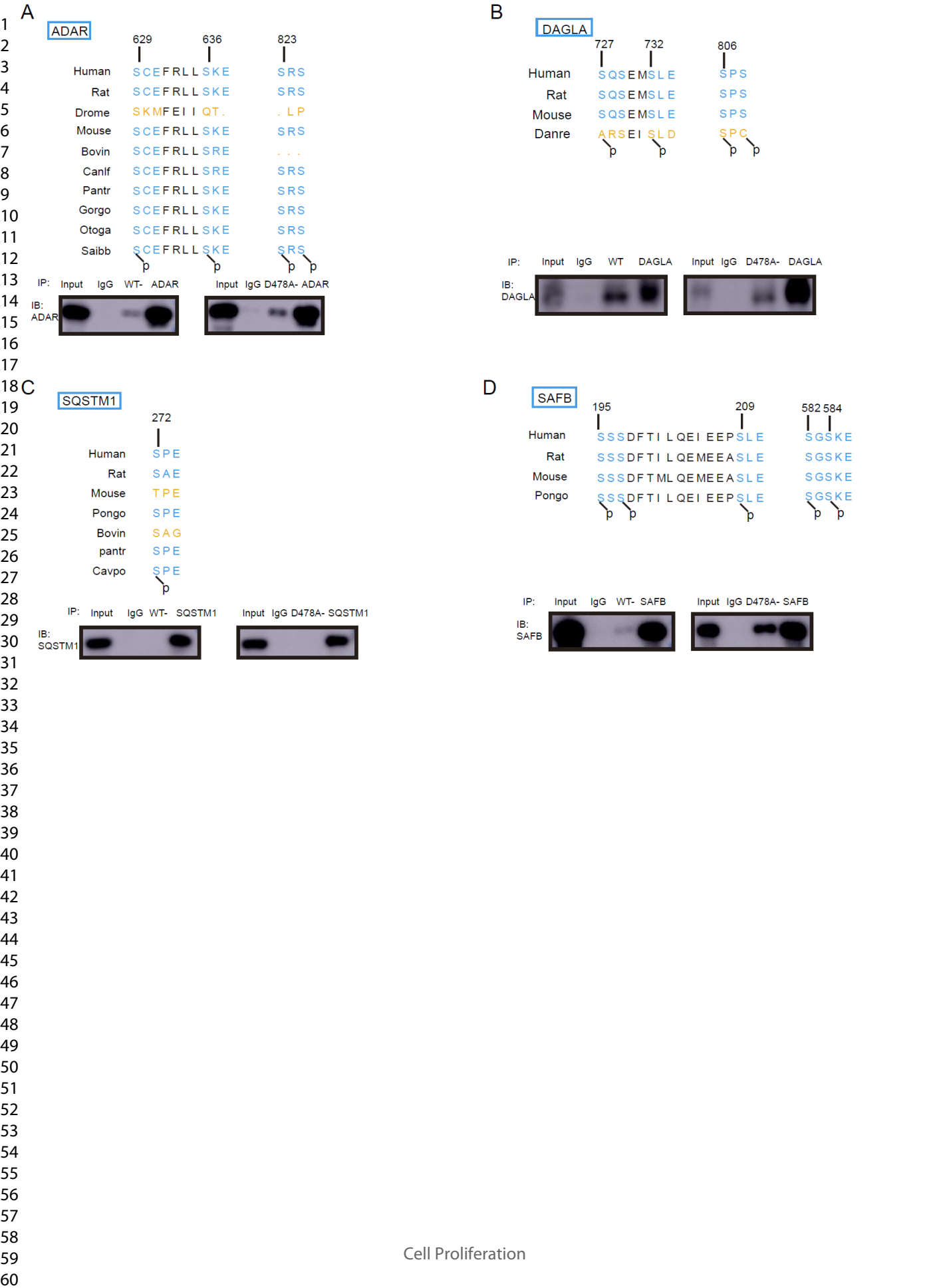

Supplement: Supplementary file 1 — Figure S1. Altered levels of CP, APOE and AFP in HIE patients and HIBD model rats. (A) Weighted gene co‐expression network analysis and principal component analysis implicate CP, APOE, PTK2B and AFP as prospective biomarkers. (B) Utilising a HIBD rat model, ELISA assays conducted after 24 h of hypoxia reveal significant fluctuations in serum CP, APOE and AFP levels. The hypoxic time for Moderate HIBD and Severe HIBD are 1.5 and 2.5 h, respectively. Data are reported as mean ± SE, from n = 4 independent biological replicates. One‐way ANOVA p‐values are reported. Figure S2. Confirmation of Proteins Interacting with FAM20C. After gel strip proteomics, ADAR, DAGLA, SQSTM1 and SAFB emerge as potential FAM20C interfactors. Subsequent homology analysis and validation of protein interactions were undertaken. (A) ADAR possess three putative phosphorylation sites (S629, S636, S823). Co‐transfection of ADAR with either wild‐type FAM20C or the D478A‐FAM20C mutant variant into HEK293T cells followed by Co‐IP confirms their interaction. (B) DAGLA, with phosphorylation sites at S727, S732 and S806, similarly interacts with FAM20C upon co‐transfection and Co‐IP assay in HEK293T cells. (C) SQSTM1, featuring a single phosphorylation site at S272, does not exhibit interaction with FAM20C. (D) SAFB, bearing three phosphorylation sites analogous to those in ADAR, is confirmed to interact with FAM20C through Co‐IP assays upon co‐transfection in HEK293T cells. [file CPR-59-e70073-s001.pdf]
